# Tumor Control After Radiosurgery in Sporadic and Neurofibromatosis Type 2 Vestibular Schwannomas

**DOI:** 10.1002/cam4.71480

**Published:** 2025-12-26

**Authors:** So Young Ji, Young‐Hoon Kim, Won Seok Chang, Ho Kang, Jung‐Il Lee, Jung Ho Han, Chae‐Yong Kim, Jong Hyun Kim, Hae Won Roh, Jeong‐Hyun Hwang, Seong‐Hyun Park, Young‐Cho Koh, Joon Cho, Seok Keun Choi, Chang Kyu Park, Se‐Hyuk Kim, Tae Hoon Roh, Sang Ryul Lee, Sang‐Won Lee, Soon‐Ki Sung, Moo Seong Kim, Won Hee Lee, Sun‐Il Lee, Seon‐Hwan Kim, Sae Hun Kim, Kyung Hwan Kim, Jung‐Won Choi, Ho Jun Seol, Young Hyun Cho, Junhyung Kim, Hyun Ho Jung, Jong Hee Chang

**Affiliations:** ^1^ Department of Neurosurgery Seoul National University Bundang Hospital Seongnam‐si South Korea; ^2^ Department of Neurological Surgery Asan Medical Center, University of Ulsan College of Medicine Seoul Korea; ^3^ Department of Neurosurgery Severance Hospital Seoul South Korea; ^4^ Department of Neurosurgery Samsung Medical Center Seoul South Korea; ^5^ Department of Neurosurgery Korea University Guro Hospital Seoul South Korea; ^6^ Department of Neurosurgery Kyungpook National University Hospital, Kyungpook National University School of Medicine Daegu South Korea; ^7^ Department of Neurosurgery KonKuk University Medical Center Seoul South Korea; ^8^ Department of Neurosurgery Kyung Hee University Medical Center Seoul South Korea; ^9^ Department of Neurosurgery Ajou University Medical Center Suwon South Korea; ^10^ Department of Neurosurgery Pusan National University Yangsan Hospital Pusan South Korea; ^11^ Department of Neurosurgery Inje University Busan Paik Hospital Pusan South Korea; ^12^ Department of Neurosurgery Inje University Haeundae Paik Hospital Pusan South Korea; ^13^ Department of Neurosurgery Chungnam National University Hospital Daejeon South Korea

**Keywords:** neurofibromatosis type 2, radiosurgery, tumor control, vestibular schwannoma, Wishart phenotype

## Abstract

**Introduction:**

The tumor control rate after stereotactic radiosurgery (SRS) for neurofibromatosis type 2‐associated vestibular schwannomas (NF2‐VSs) compared to sporadic vestibular schwannomas (S‐VSs) remains unclear. This nationwide, multicenter, retrospective study (KGKRS‐21‐001) aimed to clarify this issue.

**Methods:**

A total of 4718 patients treated with SRS for vestibular schwannomas were analyzed from 13 nationwide institutions in Korea. NF2‐VS cases were propensity score‐matched with S‐VS cases at a ratio of 1:1, based on age, tumor volume, and marginal dose, resulting in 122 cases in each group.

**Results:**

No significant differences in age, tumor volume, or marginal dose were observed between the matched cohorts. The overall tumor control rates at 1, 3, and 10 years after SRS were 93.3%, 87.7%, and 80.7%, respectively, with no significant difference between NF2‐VS and S‐VS groups (*p* = 0.63). Subgroup analysis showed that age ≤ 19 years was a significant negative prognostic factor for tumor control in NF2‐VS patients (*p* < 0.001), whereas no such correlation was found in the S‐VS cohort (*p* = 0.78).

**Conclusions:**

SRS provides comparable tumor control for NF2‐VSs and S‐VSs. However, among NF2‐VS patients, younger age (≤ 19 years) was associated with poorer tumor control, suggesting that age may be a critical factor in treatment decisions.

## 1 Introduction

Neurofibromatosis type 2 (NF2) is a genetic disorder that affects the nervous system caused by mutations in the *NF2* gene located on chromosome 22 [1, 2]. It is inherited in an autosomal dominant pattern with an incidence of approximately 1 in 50,000 [1, 3]. Despite being first described in 1822 by Wishart [4], much remains unknown about the natural history of NF2‐related tumors and manifestations. NF2 is clinically characterized by the development of multiple tumors in the cranial, spinal, or peripheral nerves, as well as meningiomas and ependymomas.

The most common manifestation of NF2 is the development of bilateral vestibular schwannoma (VSs), which can lead to bilateral hearing loss, facial palsy, and brainstem compression. The treatment of NF2‐associated VSs (NF2‐VSs) can be challenging, as the natural course of the disease is not well understood, and the treatment of bilateral VSs requires careful consideration to preserve cochlear nerve function. Recent studies have indicated that stereotactic radiosurgery (SRS) is a safe and effective treatment option for NF2‐VSs, particularly for small to medium‐sized tumors [5, 6, 7].

However, the effectiveness of SRS in terms of tumor control in patients with NF2‐VSs compared with those with sporadic vestibular schwannomas (S‐VSs) has been debated [8, 9]. Studies have shown a significant association between NF2‐VSs and tumor recurrence, whereas others have suggested that there is no significant difference in tumor control rates between NF2‐VSs and S‐VSs. However, small sample sizes limited the reliability and statistical power of these studies. To address this issue, the Korean Gamma Knife Radiosurgery Society conducted a nationwide multicenter retrospective study (KGKRS‐21‐001) using propensity score matching (PSM) to provide more robust evidence on the efficacy of SRS in patients with NF2‐VSs than in those with S‐VSs.

## 2 Materials and Methods

### 2.1 Study Population and Propensity Score Matching

This study collected the data of 4718 patients who had undergone SRS for VSs across 13 institutes nationwide, after obtaining approval from each institutional ethical committee. After excluding patients with less than 6 months of follow‐up, missing clinical data, prior radiotherapy before SRS, or who received fractionated SRS, the study included 4231 VS patients. Propensity scores, based on age, tumor volume, and marginal prescription dose of SRS, were used to match 133 (3.1%) NF2‐VS patients with S‐VS patients. The matching was performed without replacement at a ratio of 1:1, using a caliper of 0.2. Consequently, a total of 244 patients were enrolled in this study, with 122 patients assigned to each group. The overall tumor control rate was analyzed in this matched cohort. Subsequently, a subgroup analysis was performed for patients who were followed up for at least 24 months, considering the transient volume expansion that can occur in VSs after SRS and typically resolves within the first 2 years [10]. Standardized mean differences (SMDs) of covariates between the groups were calculated after matching to assess the level of balance.

### 2.2 Radiosurgery and Follow‐Ups

SRS was performed using the Leksell Gamma Knife across all participating centers. The treatment plan was generated using the Leksell Gamma Plan system (Elekta Instrument AB, Stokcholm, Sweden), which uses thin‐slice magnetic resonance (MR) imaging and/or computed tomography scanning. Target volumes were determined based on T1‐weighted, 3‐dimensional, multiplanar, rapid‐acquisition, gradient‐echo MR images obtained before and after gadolinium enhancement. In this study, the marginal prescription dose was determined by considering factors such as tumor volume and patient hearing status. Follow‐up MR imaging was typically conducted 3–6 months after SRS and then annually, and tumor volumes were measured on follow‐up MR images usually with 1 mm thickness. In this study, the slice thickness of the MR images was mainly 1 mm; however, if applied uniformly across institutions, a thickness of up to 3 mm was allowed.

### 2.3 Outcomes and Statistical Methods

The primary outcome was tumor control after SRS, which was defined as a tumor volume less than 120% of the volume at the time of SRS and/or a decrease in tumor volume after post‐radiosurgery transient volume expansion, considering the usual course of VSs after SRS [11, 12]. Tumor control rates were analyzed using the Kaplan–Meier method, and the log‐rank test was performed to compare the difference in tumor control between the matched NF2‐VS and S‐VS cohorts. To elucidate the risk factors for tumor growth, three covariates (age, marginal prescription dose, and tumor volume) were categorized and analyzed using the Cox proportional hazard model. This analysis was performed on the matched patients, who were followed up for more than 24 months. Multivariate Cox regression was performed using covariates that showed significant hazard ratios in univariate Cox regression. Cutoffs of covariates were calculated using the maximally selected rank statistics between the 2.5% and 97.5% quantile of variables based on the ‘maxstat’ package of R. If no significant cutoff was determined from the maximally selected rank statistics, a clinically meaningful cutoff was selected. This analysis using the categorized covariates was also performed for each NF2‐VS and S‐VS cohort. Statistical significance was set at *p* < 0.05. All statistical analyses were performed using R version 4.4.1 (Foundation for Statistical Computing, Vienna, Austria).

### 2.4 Ethics Statement

This study was approved by the Institutional Review Board of Seoul National University Bundang Hospital (No. B‐2203‐743‐104) as well as the Institutional Review Boards of the other participating institutions. This study was conducted in compliance with the Declaration of Helsinki. Informed consent was waived by the Board due to the retrospective study design.

## 3 Results

Before PSM, the NF2‐VS cohort significantly showed younger age (37.4 ± 17.3 vs. 55.0 ± 12.9 years, *p* < 0.001), larger tumor volume (4.4 ± 5.7 vs. 2.6 ± 3.4 cm^3^, *p* < 0.001), and lower marginal prescription dose (12.3 ± 1.2 vs. 12.6 ± 1.1 Gy, *p* = 0.018) than the S‐VS cohort. However, after PSM, these three covariates were balanced, resulting in similar covariate profiles in both cohorts. Table 1 summarizes the covariates before and after the PSM.

**TABLE 1:** Baseline characteristics and treatment parameter data of patients.

Variables	Patients, no.							
	Before PSM (*n* = 4231)				After PSM (*n* = 244)			
	NF2‐VSs	S‐VSs	*p*	SMD	NF2‐VSs	S‐VSs	*p*	SMD
Number of patients	115^a^	4099			105^b^	122		
Number of tumors	132	4099			122	122		
Female	67 (58.3%)	2476 (60.4%)	0.714	0.044	63 (60.0%)	63 (51.6%)	0.259	0.169
Prior surgery	22 (18.6%)	698 (18.9%)	1.000	0.006	25 (20.5%)	36 (29.5%)	0.139	0.209
Age, years	37.4 ± 17.3	55.0 ± 12.9	< 0.001	1.323	39.2 ± 16.9	40.1 ± 16.0	0.696	0.055
Tumor volume, cm^3^	4.4 ± 5.7	2.6 ± 3.4	< 0.001	0.387	3.6 ± 3.7	3.9 ± 3.9	0.468	0.083
Marginal prescription dose, Gy	12.3 ± 1.2	12.6 ± 1.1	0.018	0.195	12.4 ± 1.3	12.3 ± 0.9	0.659	0.057
Follow‐up duration, months	—	—	—	—	70.9 ± 68.8	61.9 ± 49.5	0.240	0.150

*Note:* All continuous variables were presented as mean ± standard deviation according to the normality tested by Kolmogorov–Smirnov test.

Abbreviations: NF2‐VSs, neurofibromatosis type 2 associated vestibular schwannomas; PSM, propensity score matching; SD, standard deviation; SMD, standardized mean differences; S‐VSs, sporadic vestibular schwannomas.

^a^ For 17 NF2 patients, bilateral NF2‐VSs were treated with SRS.

^b^ For 17 NF2 patients, bilateral NF2‐VSs were treated with SRS.

**FIGURE 1:**
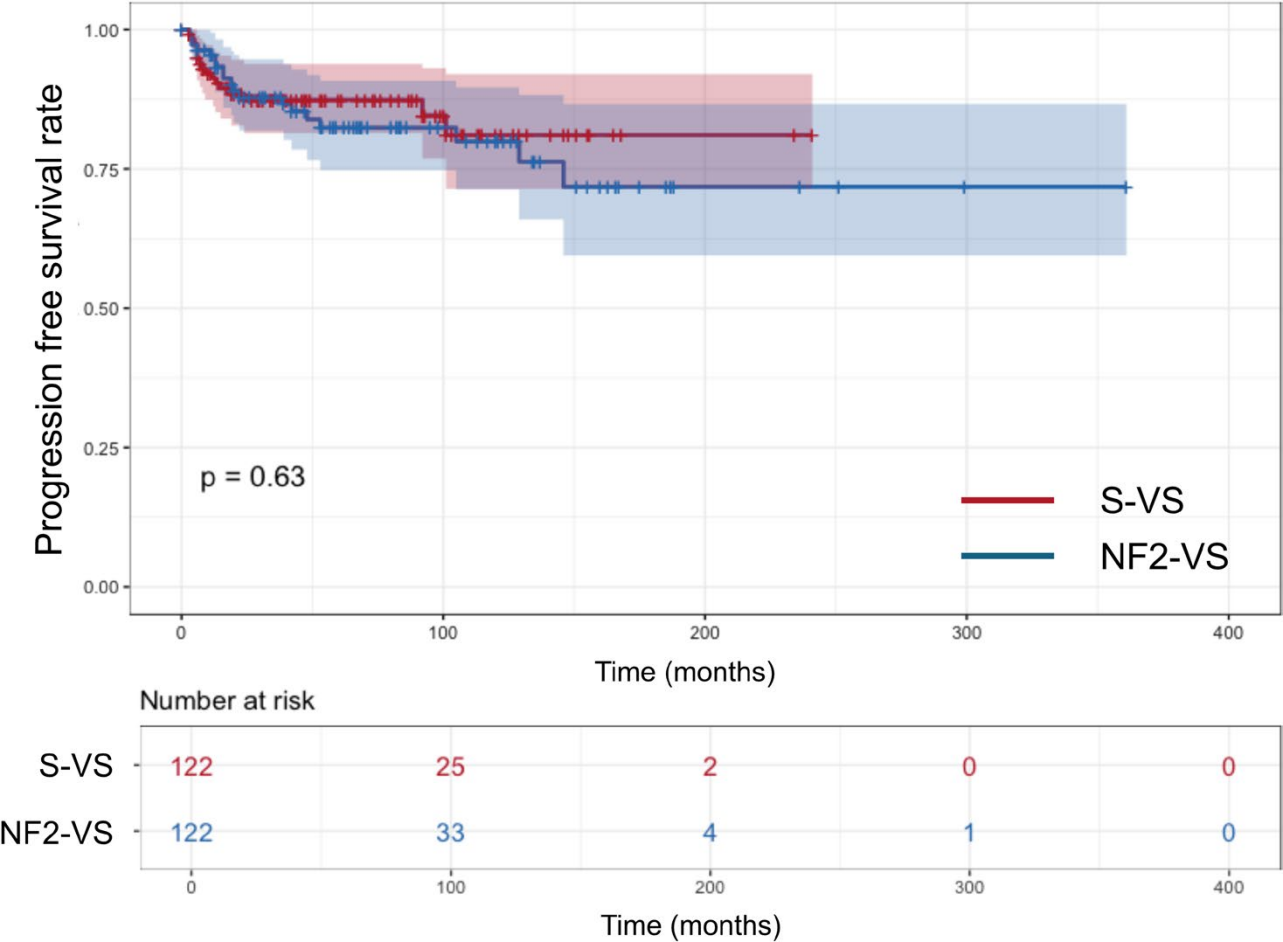
Overall Kaplan–Meier survival plot of the matched cohort for tumor progression according to NF2 (*n* = 244). *p*‐Value was calculated by log‐rank test. NF2‐VS, neurofibromatosis type 2‐related vestibular schwannoma; S‐VS, sporadic vestibular schwannoma.

The overall matched cohort analysis showed that the tumor control rates at 1, 3, and 10 years after SRS were 93.3%, 87.7%, and 80.7%, respectively. In the matched S‐VS cohort, the actual tumor control rates were 91.3%, 87.4%, and 81.1% at 1, 3, and 10 years, respectively. In contrast, the matched NF2‐VSs cohort had tumor control rates of 95.4%, 88.1%, and 79.9% at 1, 3, and 10 years, respectively. However, the difference in tumor control rates between the two matched cohorts was not statistically significant (*p* = 0.630; log‐rank test) (Figure 1).

**FIGURE 2:**
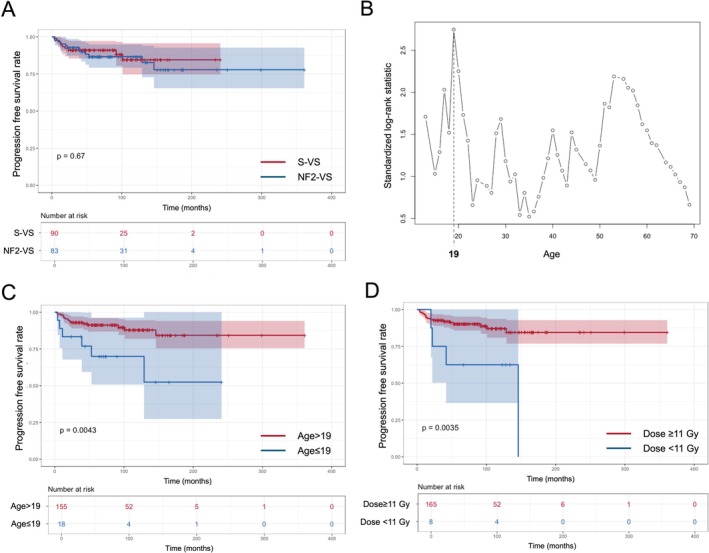
Kaplan–Meier progression plots of the matched patients who were followed for more than 24 months (*n* = 173). (A) Overall survival plot. (B) Optimal cutoff of age to categorize using maximally selected rank statistics. (C) Progression survival plot according to age that was categorized with the cutoff of 19 years. (D) Progression survival plot according to the marginal prescription dose that was categorized with the cutoff of 11 Gy. *p*‐Value was calculated by log‐rank test. NF2‐VS, neurofibromatosis type 2‐related vestibular schwannoma; S‐VS, sporadic vestibular schwannoma.

A total of 173 matched patients, including 90 S‐VS and 83 NF2‐VS patients, were followed up for more than 24 months. There was no significant difference in tumor control between the S‐VS and NF2‐VS cohorts (*p* = 0.670; log‐rank test) (Figure 2A). The maximally selected rank statistics identified a significant age cutoff of 19 years, while no significant cutoffs were found for other covariates (Figure 2B). The following selected cutoffs were used to categorize covariates other than age: 11 and 12 Gy for marginal prescription dose and 4 and 10 cm^3^ for tumor volume. Hazard ratios (HRs) for tumor control are presented in Table 2. The multivariate Cox proportional hazard model revealed that younger age (≤ 19 years) (HR, 4.54; 95% confidence interval [CI], 1.70–12.11; *p* = 0.003) and lower marginal prescription dose than 11 Gy (HR [95% CI], 5.92 [1.91–18.39]; *p* = 0.002) significantly increased the risk of tumor growth. Survival differences for tumor progression according to age and marginal dose were plotted in Figure 2C,D.

**TABLE 2:** The results of Cox proportional models for tumor control (*n* = 173).^a^

Variables	Patients, no.	Univariate		Multivariate	
		HR (95% CI)	*p*	HR (95% CI)	*p*
Female	101	0.82 (0.39–1.89)	0.649		
NF2	85	1.24 (0.54–2.85)	0.606		
The younger (≤ 19 years)	18	3.60 (1.41–9.22)	0.008	4.54 (1.70–12.11)	0.003
Marginal prescription dose < 11 Gy	8	4.39 (1.48–13.00)	0.008	5.92 (1.91–18.39)	0.002
Marginal prescription dose < 12 Gy	19	2.54 (0.93–6.91)	0.068	—	—
Tumor volume ≤ 4 cm^3^	116	0.93 (0.39–2.22)	0.866	—	—
Tumor volume ≤ 10 cm^3^	160	0.54 (0.16–1.83)	0.325	—	—
Prior surgery	38	0.75 (0.26–2.22)	0.607		

Abbreviations: CI, confidence interval; HR, hazard ratio; NF2, neurofibromatosis type 2.

^a^ A total of 173 matched patients followed more than 24 months were analyzed.

**FIGURE 3:**
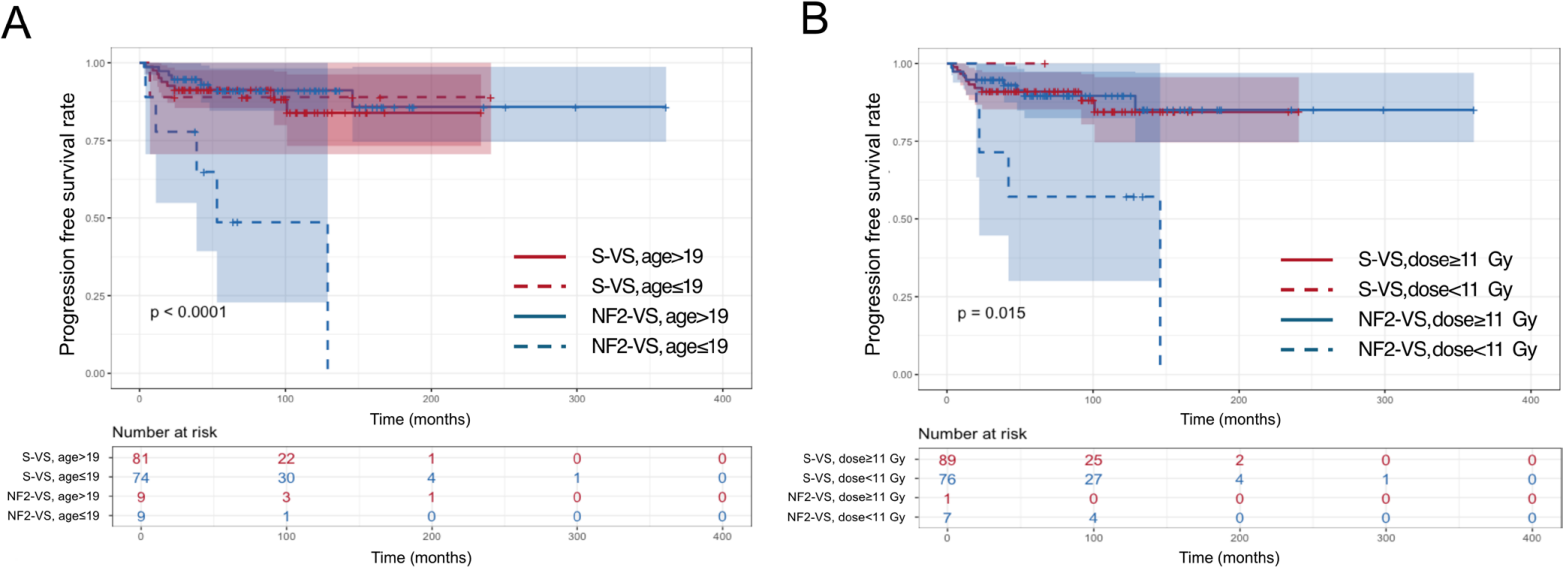
Comparison of tumor progression according to significant categorized covariates and NF2. (A) Survival plot and hazard ratio according to NF2 and age ≤ 19. (B) Survival plot and hazard ratio according to NF2 and marginal dose < 11 Gy. *p*‐Value for the comparison among 4 survival plots was calculated by log‐rank test. CI, confidence interval; HR, hazard ratio; NF2‐VS, neurofibromatosis type 2‐related vestibular schwannoma; S‐VS, sporadic vestibular schwannoma.

Survival plots according to the two significant covariates from the Cox regression, marginal prescription dose and age, each stratified by NF2 status, were presented in Figure 3. NF2‐VS in younger patients (≤ 19 years) showed significantly faster progression compared to NF2‐VSs (HR [95% CI], 9.03 [2.73–29.85]; *p* < 0.001) or S‐VSs (HR [95% CI], 6.47 [2.15–19.42]; *p* < 0.001) in adults over 19 years of age (Figure 3A and Table 3). NF2‐VSs treated with a dose lower than 11 Gy had significantly worse progression outcomes compared to S‐VSs (HR [95% CI], 4.63 [1.45–14.84]; *p* = 0.010) or NF2‐VSs (HR [95% CI], 5.76 [1.73–19.17]; *p* = 0.004) treated with doses of 11 Gy or higher (Figure 3B and Table 4). Because all NF2‐VS patients under the age of 19 were treated with a marginal dose of 11 Gy or higher, potential confounding between age under 19 and dose below 11 Gy in the NF2‐VS cohort could be excluded.

**TABLE 3:** Hazard ratios for tumor progression according to NF2 and age ≤ 19.

Group	MST, months	HR (95% CI)	*p*
S‐VS, age > 19	—	1	—
S‐VS, age ≤ 19	—	0.86 (0.11–6.86)	0.888
NF‐VS, age > 19	—	0.73 (0.27–1.98)	0.542
NF‐VS, age ≤ 19	53	6.47 (2.15–19.42)	< 0.001
S‐VS, age ≤ 19	—	1	—
NF‐VS, age > 19	—	0.81 (0.11–7.38)	0.927
NF‐VS, age ≤ 19	53	7.83 (0.88–69.28)	0.064
NF‐VS, age > 19	—	1	—
NF‐VS, age ≤ 19	53	9.03 (2.73–29.85)	< 0.001

Abbreviations: CI, confidence interval; HR, hazard ratio; MST, median survival time; NF‐VS, neurofibromatosis type 2‐related vestibular schwannoma; S‐VS, sporadic vestibular schwannoma.

**TABLE 4:** Hazard ratios for tumor progression according to NF2 and marginal dose < 11 Gy.

Group	MST, months	HR (95% CI)	*p*
S‐VS, dose ≥ 11 Gy	—	1	—
S‐VS, dose < 11 Gy	—	—	0.997
NF‐VS, dose ≥ 11 Gy	—	0.87 (0.34–2.20)	0.762
NF‐VS, dose < 11 Gy	146	4.63 (1.45–14.84)	0.010
S‐VS, dose < 11 Gy	—	1	—
NF‐VS, dose ≥ 11 Gy	—	—	0.998
NF‐VS, dose < 11 Gy	146	—	0.998
NF‐VS, dose ≥ 11 Gy	—	1	—
NF‐VS, dose < 11 Gy	146	5.76 (1.73–19.17)	0.004

Abbreviations: CI, confidence interval; HR, hazard ratio; MST, median survival time; NF‐VS, neurofibromatosis type 2‐related vestibular schwannoma; S‐VS, sporadic vestibular schwannoma.

## 4 Discussion

This study aimed to compare the efficacy of SRS between patients with NF2‐VSs and those with S‐VSs. The results showed no difference in tumor control between NF2‐VSs and S‐VSs after SRS. In NF2‐VSs, SRS is typically conducted using a lower marginal prescription dose as compared with that in S‐VSs. This adjustment aims to preserve hearing function as much as possible. The differences in the SRS parameters may have resulted in the different efficacies of SRS for NF2‐VSs compared with S‐VSs. However, as NF2 is a rare genetic disorder, most previous studies analyzed a small number of patients, leading to inconsistent results. To the best of our knowledge, this is the first nationwide multicenter study comparing NF2‐VS with S‐VS, incorporating PMS to balance the covariates associated with SRS between the two groups.

NF2‐VSs have been known to have a tendency for faster growth compared to S‐VSs [13, 14, 15]. However, in this study, there was no significant difference in tumor control between NF2‐VSs and S‐VSs after SRS. This suggests that despite the tendency for a more rapid natural growth in NF2‐VSs, SRS can still be an effective treatment option. Also, according to our findings, SRS was more commonly performed for NF2‐VSs than for S‐VSs when tumors reached a larger size. This may be due to the presence of bilateral VSs, which require careful monitoring of the hearing status and the potential for tumor size increase on both sides. When hearing declines or tumors reach a critical size, treatment becomes imperative, and SRS may become necessary.

One of key points of this study is that a younger age (≤ 19 years) was significantly associated with poor tumor control. Similarly, shortened progression‐free survival of younger patients was observed in a study with limited sample size, wherein no definitive conclusions could be drawn [16]. Historically, NF2 has been classified into two clinical forms, the Wishart phenotype and the Feiling‐Gardner phenotype [17]. The Wishart phenotype is more aggressive and is characterized by rapidly progressing multiple tumors in patients younger than 20 years of age. On the other hand, the Feiling‐Gardner phenotype is milder and presents with fewer slow‐growing tumors that arise later in life [18]. The severity spectrum of NF2 is predominantly dependent on the type of alteration present in the *NF2* gene, with truncating alterations leading to more severe disease manifestation, whereas missense loss‐of‐function mutations tend to result in a milder disease course [19, 20, 21]. The phenotypic distinction among patients with NF2 can have an impact on the effectiveness of tumor control following SRS. Considering these classifications, the results of the previous studies, and our findings, it becomes evident that NF2‐VSs in patients younger than 19 years tend to exhibit poorer tumor control with SRS compared with that in older patients. Additionally, patients with NF2 who have severe *NF2* gene alterations or the Wishart phenotype may experience poorer outcomes after SRS. Alternative radiosurgery approaches, such as hypofractionated SRS, which delivers a higher biologically effective dose than single‐fraction SRS, may offer improved efficacy—particularly in patients with the Wishart phenotype—given the relatively rapid tumor growth observed in this subgroup. However, this remains a hypothesis that warrants prospective validation. Targeting specific gene mutations with concurrent chemoradiotherapy or chemoradiosurgery may also serve as a promising approach for the management of these patients [22].

Given the current understanding that no clear ethnic differences have been established in the incidence of NF2 or the growth rate of VSs, this study—which was conducted exclusively in an ethnically homogeneous Korean population—may still have sufficient applicability to Western populations [23, 24, 25, 26]. Furthermore, because this study addressed only tumor control after SRS and did not include pre‐SRS growth rates or audiometric data, it is difficult to suggest appropriate pre‐treatment surveillance intervals. However, based on the findings of this study, it could be generally suggested that the first post‐SRS MRI should be performed within 6 months, followed by surveillance at 1‐ to 2‐year intervals for up to 10 years. For NF2‐VS in patients under 19 years of age, closer surveillance with more frequent MRI following SRS may be warranted, given the potential for aggressive behavior in this subgroup.

This study had several limitations. First, there were no clear criteria for tumor control after SRS for VSs because patients exhibit transient volume expansion following treatment. Generally, tumors tend to shrink again within 2 years after SRS [12, 27], although there are cases in which tumor shrinkage occurs several years later. This study observed good tumor control in patients with longer follow‐up periods, which was likely due to this phenomenon. In addition, it is unknown whether transient volume expansion in the NF2‐VSs occurs similarly to that of S‐VSs [28]. Despite the potential impact of selecting patients with a minimum of 2 years of follow‐up from the PMS‐overall cohort, the results consistently pointed toward a trend of early tumor control failure among patients with NF2‐VS who were younger than 20 years of age. Second, there were variations in tumor volume measurements, planning techniques, and follow‐up policies across institutions; however, the impact of such heterogeneity is likely minimal. It is because most institutions adhere to a consistent protocol for obtaining MR images with 1–3 mm thickness, and their planning, consistent with that applied for other benign brain tumors, aimed to cover more than 97% of the tumor volume. Therefore, variability between institutions was unlikely to have a significant impact on the overall results. Third, the presence of severe genetic mutation in the younger patients was not confirmed. However, confirming the presence of genetic mutations in patients with NF2 remains challenging. This is mainly due to the fact that SRS is often the primary surgery‐free treatment, and currently only 25%–60% of patients with NF2 who do not have a family history of the disease can have their genetic mutations detected in the blood [29]. Therefore, additional efforts are necessary to identify genetic mutations and develop precision radiation oncology utilizing genetic differences between the patient tumors. Also, hearing was excluded from the outcome analysis because complete audiometric data could not be reliably obtained due to the study's retrospective, multi‐institutional design. As this study focused on tumor control, we plan to conduct a follow‐up investigation specifically examining hearing preservation after SRS.

## 5 Conclusions

There was no difference in tumor control between patients with NF2‐VSs and S‐VSs after SRS. However, among the NF2‐VS group, significantly poorer tumor control was evident among patients younger than 20 years of age after SRS compared to older patients.

## Author Contributions

Acquisition, analysis, or interpretation of data: All authors Drafting of the manuscript: S.Y.Ji, Y.‐H.Kim, W.S.Chang, and H.Kang Critical revision of the manuscript for important intellectual content: All authors Statistical analysis: S.Y.Ji and H.Kang Supervision: J.H.Han and J.‐I.Lee.

## Funding

This study was supported by Korean Brain and Spinal Cord Research Foundation KBSCR‐022‐001.

## Conflicts of Interest

The authors declare no conflicts of interest.

## Supporting information


**Data S1:** cam471480‐sup‐0001‐DataS1.docx.

## Data Availability

The data are not publicly available due to the protection of private patient health information. However, the dataset generated during the analysis in the current study is available after anonymization from the corresponding author on reasonable request.
